# The genetic architecture of fitness in a seed beetle: assessing the potential for indirect genetic benefits of female choice

**DOI:** 10.1186/1471-2148-8-295

**Published:** 2008-10-26

**Authors:** T Bilde, U Friberg, AA Maklakov, JD Fry, G Arnqvist

**Affiliations:** 1 Animal Ecology/Department of Ecology and Evolution, Evolutionary Biology Centre, University of Uppsala, Uppsala SE-753 32, Sweden; 2 Department of Biological Sciences, Ecology and Genetics, University of Aarhus, 8000 Aarhus C, Denmark; 3 Department of Ecology, Evolution and Marine Biology, University of California, Santa Barbara, California 93106-9610, USA; 4 School of Biological, Earth and Environmental Sciences, The University of New South Wales, Kensington, Sydney 2052, Australia; 5 University of Rochester, Department of Biology, Rochester, New York 14627-0211, USA

## Abstract

**Background:**

Quantifying the amount of standing genetic variation in fitness represents an empirical challenge. Unfortunately, the shortage of detailed studies of the genetic architecture of fitness has hampered progress in several domains of evolutionary biology. One such area is the study of sexual selection. In particular, the evolution of adaptive female choice by indirect genetic benefits relies on the presence of genetic variation for fitness. Female choice by genetic benefits fall broadly into good genes (additive) models and compatibility (non-additive) models where the strength of selection is dictated by the genetic architecture of fitness. To characterize the genetic architecture of fitness, we employed a quantitative genetic design (the diallel cross) in a population of the seed beetle *Callosobruchus maculatus*, which is known to exhibit post-copulatory female choice. From reciprocal crosses of inbred lines, we assayed egg production, egg-to-adult survival, and lifetime offspring production of the outbred F1 daughters (F1 productivity).

**Results:**

We used the bio model to estimate six components of genetic and environmental variance in fitness. We found sizeable additive and non-additive genetic variance in F_1 _productivity, but lower genetic variance in egg-to-adult survival, which was strongly influenced by maternal and paternal effects.

**Conclusion:**

Our results show that, in order to gain a relevant understanding of the genetic architecture of fitness, measures of offspring fitness should be inclusive and should include quantifications of offspring reproductive success. We note that our estimate of additive genetic variance in F_1 _productivity (*CV*_*A *_= 14%) is sufficient to generate indirect selection on female choice. However, our results also show that the major determinant of offspring fitness is the genetic interaction between parental genomes, as indicated by large amounts of non-additive genetic variance (dominance and/or epistasis) for F_1 _productivity. We discuss the processes that may maintain additive and non-additive genetic variance for fitness and how these relate to indirect selection for female choice.

## Background

Female choice is defined as when a trait in females (behavioral, morphologic, physiological) biases reproductive success among males towards certain male phenotypes over others [1]. The evolution of adaptive female mate choice relies on variation in the quality of potential mates, and the ability of females to select sires of the highest quality [2]. While direct benefits of mate choice provide females with immediate benefits in the form of resources, indirect benefits of mate choice are acquired through enhanced genetic quality of offspring [3-7]. Female choice for high quality males may result from either pre-copulatory mating biases [2] or post-copulatory processes in the female reproductive tract that lead to differential use of male gametes for fertilization [8]. Models for the evolution of female choice based on indirect genetic benefits are usually grouped into good genes models, where alleles with additive effects confer fitness benefits, and compatibility models, where the combining ability of specific alleles (i.e., epistasis) of the male and female genomes determines fitness [2,9,10]. These models, thus, rely on different types of genetic variation for fitness.

Female choice may result from indirect selection, by a process known as the "good genes" process [11]. This results from an association, by linkage disequilibrium, between alleles that determine female preference at one locus and those that affect viability at other loci. Theoretical models have shown that such associations develop largely as a result of additive effects of viability loci [12]. Epistasis contributes little if anything to this process, simply because recombination tends to disassociate co-adapted alleles across viability loci [12]. Similarly, good genes models that have considered dominance variation, in the form of recessive deleterious mutations, show that dominance have little if any effect on the good genes process [13,14].

Genetic compatibility scenarios are based on females deriving indirect genetic benefits from pairing with genetically compatible males [6,9,10,15]. Here, compatibility refers to cases where the fitness effects of an allele depend on either its homologue (dominance and over-dominance) or on a specific allele at another locus (epistasis) and these models rely on non-additive genetic variation across the genome [15-17]. Female choice for genetically compatible mates, thus, do not and can not involve indirect selection (see above), but would result from direct selection among females for indirect genetic benefits. We note that such processes are not expected to lead to the "run-away" selection that has been suggested to result in evolutionary exaggeration or elaboration of male traits. Further, although good genes and compatibility models for the evolution of female choice are distinct in theory, they may not be mutually exclusive in the sense that additive and non-additive genetic effects are not statistically independent [16].

For an association between female preference genes and viability/fitness genes to develop, sufficient levels of additive genetic variation for fitness must be present in the population. The classical interpretation of Fisher's fundamental theorem [18] holds that additive genetic variation for fitness is effectively depleted by directional selection [16,19]: alleles conferring fitness advantages should become fixed in the population while those that reduce fitness should gradually be lost [20,21]. The evolvability of fitness should therefore be very low in a stable environment. This general scenario has been supported by numerous studies of heritability, collectively suggesting that the heritability of traits closely related to fitness (e.g. survival and fecundity) tends to be lower than that of morphological and behavioral traits, which generally are less closely associated with fitness [16,22-24]. However, the narrow sense heritability (*h*^2^) of a trait represents the ratio of additive genetic variance (*V*_*A*_) to the total phenotypic variance (*V*_*P*_) and the heritability of traits largely affected by non-additive effects or environmental factors will thus by definition be low [25-28]. Heritability estimates may therefore often be inappropriate for quantifying the long-term evolutionary potential of a trait (see discussion in [21]) and they are entirely inadequate for detecting epistatic interactions. Instead, Houle (1992) argued that the coefficient of additive genetic variance (*CV*_*A*_), where additive genetic variance is scaled by the trait mean, is a more appropriate measure when comparing the evolutionary potential of quantitative traits. When comparing *h*^2 ^and *CV*_*A *_across traits in *Drosophila*, Houle (1992) found that *CV*_*A *_for fitness-related traits were actually higher than those for morphological traits (see also [29] for a similar finding). One obvious explanation for this is the fact that traits closely related to fitness should be more polygenic and should thus capture genetic variation across many loci [30]. Fitness related traits also show a high mutational input to genetic variance [31], again presumably reflecting their polygenic nature [32]. The same scaling can be applied to other components of genetic variance (e.g. non-additive components [D. Houle, pers. comm.]).

Non-additive genetic effects caused by interactions among alleles within and between loci (i.e., dominance and epistasis) may be important determinants of fitness [28,33-35]. Given that quantitative fitness traits are polygenic, we would expect that fitness related traits would harbor fairly high absolute levels of non-additive genetic variation [21,26,36,37].

The general importance of indirect genetic benefits for the evolution of female choice is still debated [11-13,38]. Further, the relative roles of the good genes process and genetic compatibility scenarios remain largely unexplored [6,11,15,17]. One way to address these questions is to partition and quantify additive and non-additive genetic variance for fitness, since the nature of the potential indirect genetic benefits of mate choice depends critically on the genetic architecture of fitness. While additive genetic variance of fitness has been studied in a few populations of birds and mammals [22,26,39,40] data on the genetic architecture of fitness or fitness related traits are scarce [41-44].

The purpose of this study was to investigate the genetic architecture of fitness, with the specific goal of estimating additive and non-additive (composite of dominance and epistasis) genetic variance components. This was achieved by employing a quantitative genetic design, the diallel cross [34,35,45], that allows additive and non-additive genetic variance components to be estimated using the bio model [41,45-49]. As a model system, we used the seed beetle *Callosobruchus maculatus *(Coleoptera, Bruchidae). This species is moderately polyandrous and is known to exhibit post-copulatory female choice [50-54]. The species is also well suited for establishing the homozygous discrete genotypes necessary for conducting the reciprocal cross [34]. We first created inbred lines, with an estimated inbreeding coefficient of 0.89, from a large and outbred population. In reciprocal crosses of these genotypes, we then assayed egg production, egg-to-adult survival and lifetime offspring production of the F1 daughters. Hence, we obtained integrative measures of offspring quality that reflect the net effect of the parental genetic contribution to offspring fitness. Such data are scarce but vital for understanding the potential fitness consequences of mate choice [7,55].

## Methods

Our study animal was a laboratory population of *C. maculatus *(F.) (Coleoptera: Bruchidae), obtained from C.W. Fox, University of Kentucky, Lexington. This population originated from infested mung beans (*Vigna radiata*) collected in Tirunelveli, India in 1979 [56], and has been maintained in the laboratory for more than 100 generations at population sizes > 1500 individuals. Hence, this population is unlikely to have gone through a bottleneck that would have depleted the genetic variation [56,57] (see also [58]). In addition, this *C. maculatus *population was well suited because females eliminate competition among progeny within host beans by laying only one egg on each bean [56,57,59].

Young *C. maculatus *larva bores into the bean where it completes development, emerging as an adult after 24–26 days at a temperature of 25°C. We kept beetles on organically grown Mung beans in incubators at 25°C and a relative humidity of approx. 60%. Virgin beetles for our experiments were obtained by isolating individual beans containing single larvae/pupae prior to eclosure. Inbred lines were founded in December 2004, in the following manner. Virgin females (N = 215) were mated with one male each and then placed individually in vials (30 ml) containing more than 100 Mung beans for oviposition. Prior to eclosure, beans containing single larvae were isolated and a single virgin female was mated once to a full sib brother and then confined individually in a container (30 ml) with an excess of Mung beans for oviposition. This procedure was repeated for 10 generations. The estimated inbreeding coefficient after 10 generations of full sib matings is *F *= 0.89.

Within each line and each generation, the above procedure was performed in parallel using two females each mated to one full sib brother to reduce the risk of stochastic reproductive failure and hence reduce the rate of line loss. Nonetheless, approximately 60% of the lines were lost during the inbreeding process. This line loss represents a source of selection that could potentially bias the estimates of genetic variation. The likely effect would be to deflate genetic variance estimates, which would render our variance estimates conservative. To increase the variance estimates, the selection process would have had to cause selective extinction of lines with intermediate breeding values, leaving lines with high and low values, which seems unlikely.

### The diallel reciprocal cross

From a set of 19 lines we employed a full diallel design with reciprocal crosses [34,45]. From each line, we isolated virgin males and females and then paired females from each line with males from each of the 19 lines. Each focal pair was placed in a vial with excess beans as described above. Preliminary observations suggested that virgin females accepted the first mating regardless of male genotype, hence, we assume here that all pairs would mate. In total, we performed 361 line crosses (342 between-line crosses and 19 within-line crosses). Between-line crosses were each replicated three (in some cases four) times (N = 3(4) focal pairs per cross) and within-line crosses were replicated four times (N = 4 focal pairs per cross). From each focal pair, we recorded the following four variables: (1) the total number of eggs produced (lifetime egg production); (2) the total number of adult offspring produced (lifetime offspring production); and (3) the egg-to-adult survival of offspring, i.e. juvenile fitness (number of offspring produced divided by the number of eggs laid). Total offspring production was obtained by isolating all beans with eggs until offspring eclosed. We then randomly selected 4–6 daughters from each focal pair and paired each virgin daughter with a randomly selected outbred male following the procedure described above. These males came from crosses between inbred lines (overall from 82 inbred lines, see procedure above), and males never originated from overlapping parental lines as the females. With this procedure, we yielded a measure of (4) the lifetime offspring production of F1 daughters of each focal pair (F1 productivity). With this design, we did not expect male genotype to contribute significantly to genetic variance in F1 productivity. This assumption was confirmed by statistical analyses showing that the genotype of the F1 males explained only a very small proportion of the variance (< 2%) in F1 productivity. Hence, male genotypic effects were therefore subsequently disregarded.

Unmated *C. maculatus *females produce some unfertilized eggs when a suitable substrate is available, but some females in our experiment nevertheless failed to produce eggs. For all analyses reported below, we ran parallel models using both the full data set and a data set excluding females with no egg/offspring production. Both types of data yielded very similar variance component estimates. However, because the restricted data set better conformed to the assumption of our inferential models, we present these results below (Table 1). Here, pairs that did not produce any eggs were coded as missing values. Egg-to-adult survival was transformed prior to analyses using the arcsine square root transformation.

**Table 1:** Observational variance component estimates (SE) and likelihood ratio tests of the null hypothesis H_0_: *σ*^2 ^= 0 against H_A_: *σ*^2 ^> 0.

	Lifetime egg production		Lifetime offspring production		Egg-to-adult survival (arcsine sqrt)		F1 productivity	
Variance component	Estimate (SE)	P	Estimate (SE)	P	Estimate (SE)	P	Estimate (SE)	P

*σ*^2^_n_	0	-	0	-	0.0012 (0.002)	0.12	9 (3.7)	< 0.001
*σ*^2^_t_	8.5 (4.6)	0.015	5.1 (4.3)	0.055	0.0046 (0.003)	0.035	6.7 (3.7)	0.015
*σ*^2^_m_	12.7 (5.3)	< 0.001	10.8 (4.6)	< 0.001	0.003 (0.003)	0.035	4.3 (2.7)	0.004
*σ*^2^_p_	12.6 (5.3)	< 0.001	11.5 (4.9)	< 0.001	0.0043 (0.003)	0.01	0	-
*σ*^2^_k_	0.2 (6.2)	0.5	0.5 (6)	0.18	0	-	8.5 (4.7)	0.008
*σ*^2^_rep_	125.7 (7.5)	< 0.001	121.9 (7.2)	< 0.001	0.118 (0.006)	< 0.001	24.1 (4.1)	< 0.001
*σ*^2^_w_							116.1 (3.7)	< 0.001

*σ*^2^_rep_: variance among replicate crosses

*σ*^2^_w_: variance among F_1 _females from the same replicate cross

### Variance component estimates

We estimated variance components using the bio model of Cockerham & Weir (1977) and Lynch & Walsh (1998). For the first three traits (excluding F_1 _productivity), the model equation was

(1) *Z*_*ijk *_= *μ *+ *N*_*i *_+ *N*_*j*_*+ T*_*ij *_+ *M*_*j *_+ *P*_*i *_+ *K*_*ij *_+ *R*_*k*(*ij*)_.

Here, *Z*_*ijk *_= the trait value from the *k*'th replicate cross between line *i *males and line *j *females, and *μ *= mean phenotypic value of the population. The other terms are assumed to be mutually independent, normally distributed variables with mean zero, representing the following random effects:

*N*_*i *_and *N*_*j *_= haploid nuclear contributions from parental lines *i *and *j *(independent of sex).

*T*_*ij *_= interaction between haploid nuclear contributions.

*M*_*j *_= maternal genetic and environmental effects of line *j *when used as dams.

*P*_*i *_= paternal genetic and environmental effects of line *i *when used as sires.

*K*_*ij *_= interaction between maternal and paternal effects.

*R*_*k*(*ij*) _= effect of *k*'th replicate cross within dam line × sire line combination.

For F_1 _productivity, the model equation was:

(2) *Z*_*ijkl *_= *μ *+ *N*_*i *_+ *N*_*j *_+ *T*_*ij *_+ *M*_*j *_+ *P*_*i *_+ *K*_*ij *_+ *R*_*k*(*ij*) _+ *W*_*l*(*k*(*ij*))_.

Here, Z_ijkl _is the productivity of the l'th female from the k'th replicate cross, and W_l(k(ij)) _is the residual (within replicate cross) effect of individual l.

Estimates of the variances of the terms in equation [1] and [2] (*σ*^2^_*n*_, etc.; see below for interpretations) were obtained by restricted maximum likelihood (REML) using the MIXED procedure in SAS v. 9.1.3. (SAS Institute, 2004), by expressing the covariance between families as linear functions of the variances (see [46], for details). The analysis was performed on a matrix of 342 between-line crosses, excluding all within-line crosses, as required by the bio model [34,46]. We tested the one-sided hypotheses that parameter estimates are larger than 0 with likelihood ratio tests, by comparing models where a given parameter was set to 0 with a model where all parameters were allowed to assume non-negative values [46]. For the analysis of F_1 _productivity, we included a term to separate the variance among females within replicate crosses from the variance among replicate crosses. This was done by adding the statement "RANDOM REPCROSS (SIRE*DAM);" to the PROC MIXED commands, where "SIRE" and "DAM" refer to the line of the sire and dam, and "REPCROSS" is a class variable. For the other traits there was only one measurement per replicate cross, hence the replicate cross variation is not distinguishable from the residual variation.

The "observational" variance components (denoted by sigma's) have the following interpretations in terms of "causal" components (denoted by *V*'s):

1) *σ*^2^_*n*_: nuclear additive variance. Assuming that our lines represent a random sample from the base population, and that we have estimated *F *correctly (*F *from pedigrees does not always reflect the actual *F*, due to selection for heterozygosity that can occur during the inbreeding process), *σ*^2^_*n *_= 1/2 *FV*_*A *_+ 1/4 *F*^2^*V*_*AA*_, where *V*_*A *_and *V*_*AA *_are the additive genetic and additive by additive epistatic variances, respectively, in the base population, and higher order epistasis is ignored for simplicity ([16], equation 15.8). If *V*_*AA *_is assumed to be small, then *V*_*A *_can be estimated as 2*σ*^2^_*n*_/*F*.

2) *σ*^2^_*t*_: nuclear interaction variance. Under the same assumptions as above, *σ*^2^_*t *_= 1/2 *F*^2^*V*_*AA *_+ *F*^2^*V*_*D *_+ *F*^3^*V*_*AD *_+ *F*^4^*V*_*DD*_, where *V*_*AD *_and *V*_*DD *_are the additive by dominance and dominance by dominance epistatic variances, respectively. Assuming the epistatic terms are small, the dominance variance *V*_*D *_can be estimated as *σ*^2^_*t*_/*F*^2^.

3) *σ*^2^_*m*_: maternal effect variance *V*_*m*_, including both maternal genotype and common-environment effects, as well as possible interactions or co-variances between maternal nuclear and maternal extra-nuclear effects.

4) *σ*^2^_*p*_: paternal effect variance *V*_*p*_, including both paternal genotype and common-environment effects, as well as possible interactions or co-variances between paternal nuclear and paternal extra-nuclear effects.

5) *σ*^2^_*k*_: interaction variance of paternal and maternal effects, and of nuclear and extra-nuclear effects (e.g. cyto-nuclear), *V*_*k*_.

6) *σ*^2^_*rep *_: variance among replicate crosses within line combinations.

7) *σ*^2^_*w*_: (for F_1 _productivity only) variance among females within replicate crosses.

Note that neither *σ*^2^_*rep *_or *σ*^2^_*w *_(or their sum) can be equated with the environmental variance *V*_*E*_, because unless the parental lines are fully inbred (*F *= 1), both *σ*^2^_*rep *_and *σ*^2^_*w *_will have genetic as well as environmental contributions. To estimate *V*_*E*_, we subtracted the other causal components of variance from the total phenotypic variance (*V*_*tot*_):*V*_*E *_= *V*_*tot *_- *V*_*A *_- *V*_*D *_- *V*_*M *_- *V*_*P *_- *V*_*K*_. *V*_*tot *_was estimated as the sum of all the observational components of variance, taking *σ*^2^_*n *_twice (because *N *appears twice in the model equation).

Coefficients of genetic variation (CV) for each parameter estimate were calculated using the additive genetic coefficient of variation, ${\text{CV}}_{\text{A}}=100\sqrt{{\text{V}}_{\text{A}}}/\bar{X}$[21] where $\bar{X}$ is the phenotypic trait mean. Coefficients of genetic variation allow comparisons of the sources of phenotypic variation across different traits.

## Results

The partitioning of phenotypic variance into observational genetic variance estimates is given in Table 1. In the initial diallel cross, variance in lifetime egg production came from highly significant contributions of *σ*^2^_*m *_and *σ*^2^_*p*_, representing line-specific maternal and paternal effects, respectively. The strong maternal effect is not surprising, because this term includes the effect of the female's own (inbred) genotype on her egg production. The maternal effect term could also include a contribution of common environment effects shared by different females from the same line. The highly significant paternal effect on egg production, which is of the same magnitude as the maternal effect, is more surprising. This indicates that the genotype of the inbred line males, or the environment shared by different males from the same line, strongly affected egg production by their mates. In contrast, there was no evidence for nuclear additive effects (*σ*^2^_*n*_) on egg production, and nuclear non-additive effects (*σ*^2^_*t*_), while significant, were smaller than the paternal and maternal effects. These results are not surprising, because we would not expect that the genotype of a female's eggs would have much if any direct influence on the number of eggs she produces. Finally, there was no evidence for non-reciprocal interaction effects (*σ*^2^_*k*_) on egg production. Variance components and significance levels for lifetime offspring production were similar to those for lifetime egg production (Table 1), reflecting the overall high survival in these crosses (about 80%).

The proportion surviving in the initial crosses showed no evidence for nuclear additive or non-reciprocal interaction effects, marginally significant nuclear dominance and maternal effects, and a significant paternal effect (Table 1). The latter result indicates that some property of male line influenced egg survival, independently of the males' genetic contribution to the eggs (the latter would be reflected in *σ*^2^_*n *_and/or *σ*^2^_*t*_).

Up to six F_1 _females from each initial cross were mated to randomly selected outbred males, and their productivity measured. We treat productivity of these females as a trait of the females themselves, rather than of the cross, as this allows us to investigate the co-expression of female dam and sire genotypes. This approach is justified by the fact that male genotype explained only a small fraction of the productivity variation (see Methods). We found highly significant nuclear additive variance (*σ*^2^_*n*_) for F_1 _productivity, and marginally significant nuclear non-additive variance (*σ*^2^_*t*_). While *σ*^2^_*m *_also contributed significantly to F_1 _productivity (possibly reflecting cytoplasmic effects), *σ*^2^_*p *_did not. In contrast to the results from the first cross, the non-reciprocal compatibility variance *σ*^2^_*k *_also contributed significantly to the variance in F_1 _productivity. Finally, there was a highly significant effect of initial replicate cross (*σ*^2^_*rep*_), meaning that full-sibs resembled each other in productivity more than non-full sibs from the same cross combination (line *i *female × line *j *male). This likely largely reflects common environment effects, although because the lines were not completely inbred, some genetic divergence between replicate full-sibships may have contributed as well.

**Figure 1:**
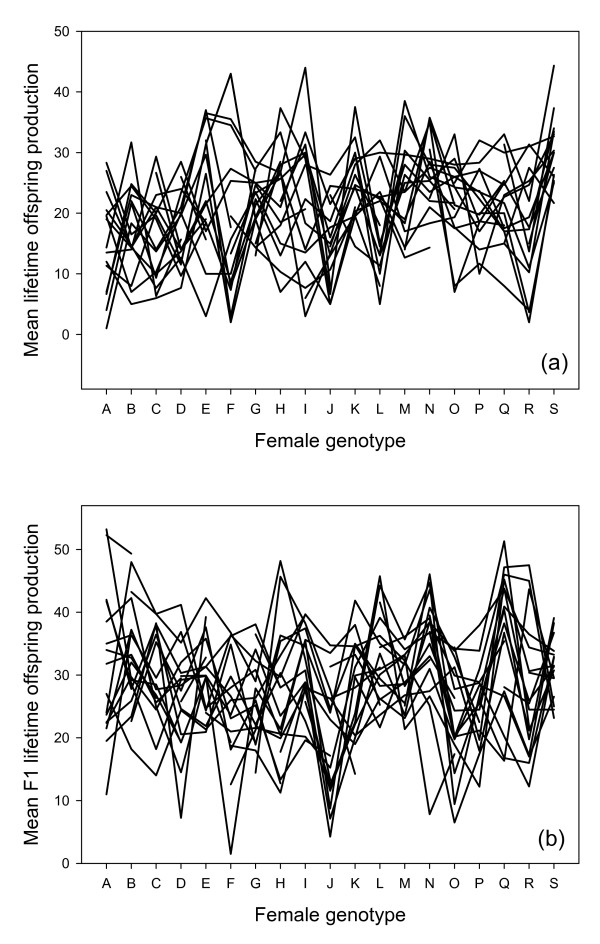
**Mean (a) lifetime offspring production and (b) mean F_1 _daughter lifetime offspring production for all combinations of female (abscissa) and male (solid lines) genotypes (within-line crosses excluded). **Note the extensive amount of interactions between genotypes.

Estimates for causal variance components and coefficients of genetic variation are listed in Table 2. The estimates are given mainly for heuristic purposes. They assume that epistatic variance is absent; if present, epistasis would contribute mostly to the estimate for dominance variance, and slightly to the additive variance. The sources of genetic variance in fitness were strikingly different when estimated in different life-history stages. Overall, variance in parental productivity and egg-to-adult survival was dominated by maternal and paternal effects. In contrast, variance in F_1 _productivity was affected by sizeable components of both additive and non-additive genetic variation as well as by sex-specific interactions between the maternal and paternal genomes. The generally strong interaction effects are illustrated in Figure 1.

**Table 2:** Estimates of raw and scaled variances and coefficients of genetic variation (CV %; see Houle 1992).

	Lifetime egg production			Lifetime offspring production			Egg-to-adult survival (arcsine sqrt)			F1 productivity		
Variance	Esti-mate	%	CV	Esti-mate	%	CV	Esti-mate	%	CV	Esti-mate	%	CV

V_A_	0	0	0	0	0	0	0.0028	2.12	4.60	20.32	11.44	14.16
V_D_	10.67	6.69	14.17	6.50	4.33	12.81	0.0058	4.37	6.60	8.45	4.75	9.13
V_M_	12.73	7.98	15.48	10.80	7.20	16.51	0.0030	2.24	4.72	4.26	2.40	6.48
V_P_	12.59	7.89	15.39	11.54	7.70	17.07	0.0043	3.26	5.70	0	0	0
V_K_	0.17	0.10	1.77	0.51	0.34	3.58	0	0	0	8.48	4.77	9.15
V_E_	123.45	77.35	48.20	120.58	80.42	55.17	0.1167	88.01	29.62	136.16	76.64	36.66

As expected, we did not detect any differences in lifetime egg production when comparing within- and between-line crosses. In contrast, we found strong evidence for heterosis (Table 3) for variables where the maternal and paternal genomes were co-expressed. Heterosis clearly affected juvenile performance, as reflected in both egg-to-adult survival as well as F_1 _productivity. We note that the latter effect, where full co-expression is expected, was stronger than the former (Table 3).

**Table 3:** Comparisons of fitness components in within- and between-line crosses (excluding crosses where no eggs where produced).

Trait	Within-line	Between-line	*F *– tests of equality of means
Lifetime egg production	23.73 (1.53)	23.05 (0.4)	*F*_1,946 _= 0.17, *P *= 0.67
Lifetime offspring production	16.07 (1.48)	19.9 (0.4)	*F*_1,950 _= 5.93, *P *= 0.015
Egg-to-adult survival (%)	62.01 (0.03)	79.75 (0.01)	*F*_1,946 _= 30.97, *P *< 0.001

F1 productivity	16.97 (0.82)	31.82 (0.25)	*F*_1,2903 _= 209, *P *< 0.001

Given are mean (SE) and *F *– tests of equality of means.

## Discussion

Our analyses revealed sizeable additive and non-additive components of genetic variation for our most comprehensive fitness variable (F_1 _productivity) in *C. maculatus*, illustrating that the diallel cross offers a useful empirical route to provide insights into rather complex aspects of the genetic architecture of fitness. In addition, we found large paternal and maternal effects indicative of direct effects on egg production. We also document striking differences in the genetic architecture of fitness traits measured in different life history stages: while egg-to adult survival was significantly affected by paternal effects, we found considerable additive and non-additive genetic variation for F_1 _productivity. This stresses the importance of obtaining comprehensive measures of fitness when assessing the potential for indirect genetic benefits. Below we discuss these findings in more detail, and use our results to discuss potential processes that may contribute to genetic variance in fitness. Finally, we ask what the implications are of our results in terms of the evolution of female choice by indirect selection.

In general, we found fairly high levels of both additive and non-additive genetic variance in F_1 _productivity. Our estimate of additive genetic variance for F_1 _productivity was 14% expressed as the coefficient of additive genetic variation *CV*_*A*_, which lies within the range of *CV*_*A *_of lifetime fitness reported from natural populations of other species (6–44% for females) [40,60,61]. This amount of genetic variation should in theory be sufficient to derive additive genetic benefits of mate choice [62] (see below). One potential concern is that our estimates may be affected by selection when creating distinct genotypes through inbreeding, possibly due to the purging of genes with non-additive effects (e.g., recessive lethals). Unless the removal of alleles with non-additive effects caused selective extinction of genotypes with intermediate breeding values, which seems unlikely, this process would tend to decrease estimates of additive genetic variation [63]. Thus, selection during the inbreeding process is unlikely to alone explain the sizeable amount of additive genetic variation for fitness found here (see Methods). Because the continual input from mutations seems unlikely to account for the presence of levels of additive genetic variation for fitness as high as those documented here [31], our study suggests that sources other than mutation contribute to additive genetic variation.

Previous studies employing reciprocal crosses/the bio model have dealt with life history traits of the parental lines (e.g. fecundity) or of the offspring, typically in the juvenile stage [41-44,64,65]. Most of these studies report low or moderate levels of additive genetic variation for traits related to parental fitness, but these results are not directly comparable to those reported here. This is because, as shown here, the genetic architecture of fitness may vary across life history traits, and it is imperative to include offspring reproductive success in any inclusive measure of fitness [66-68].

We found significant non-additive variance, *σ*^2^_*t *_suggesting that reciprocal epistatic interactions between nuclear genes and/or dominance significantly affect fitness in our population. There was also a sizeable effect of more complex and sexually non-reciprocal genetic interactions (*σ*^2^_*k*_) in F_1 _productivity. Sexually non-reciprocal epistasis may reflect cyto-nuclear genetic interactions, which has been shown to affect fitness related traits in both *C. maculatus *[69,70] and other insects [71,72]. Sexually non-reciprocal interaction could also arise as a result of *Wolbachia *infections [73,74], but we note that *Wolbachia *infections has been sought for but never found in *C. maculatus *[75].

### Parental effects

Both variance in parental environmental effects and nuclear genetic variation among parents will contribute to parental effects. In our case, however, we suggest that parental effects are largely due to genetic variation, because rearing conditions were highly standardized and thus uniform across lines. Parental effects were large for lifetime egg and offspring production but lower for egg-to-adult survival and F1 productivity (Table 1). The former measure primarily reflects variance in fertility across lines, while the latter measure will be affected by the co-expression of the two parental haploid genomes. We note here that sire families that gave rise to high egg production by their mates also gave rise to higher survival of the offspring (unpublished data), suggesting that some property of the male ejaculate (see below) influenced both egg production and survival of the eggs. These facts underscore the point that studies of the genetic architecture of fitness should focus on comprehensive measures of offspring adult performance.

Sizeable maternal effects, both genetic and environmental, are well documented in *C. maculatus *[76-78]. Furthermore, paternal effects are also pronounced in this taxa, mediated by seminal fluids in the large ejaculate (approximately 8% of male body weight) [79], and our results reflect this fact. Nutrients in the male ejaculate have been shown to be incorporated into reproductive and somatic tissue in female Bruchid beetles [80] and females that receive larger ejaculates show elevated egg production [79,81,82]. There is growing evidence that ejaculate volume shows additive genetic variation and it is often positively correlated with body size or male condition in insects [51,79,83,84]. For example, nutrient investment and spermatophore size in the butterfly *Pieris napi *is heritable, and male genotype influence female fecundity and longevity [85]. This suggests that females, by mating with males with large ejaculates, will derive direct fecundity and survival benefits as well as indirect genetic benefits. Such indirect benefits may, however, be negated by sexually antagonistic genes (discussed below).

### Implications for indirect sexual selection

The presence of additive genetic variance provides the raw material for indirect selection by the good genes process [2,3,14,17]. The amount of standing additive genetic variation for fitness present in our population should in principle be sufficient to generate indirect selection on female choice [12]. Further, genetic variation for fitness in our population was also influenced by genes with non-additive effects, and the raw material for females deriving indirect benefits by mate compatibility is thus also present. However, while non-additive genetic effects may be ubiquitous [28,86], the role of these effects in driving the evolution of mating biases is both debated [5,15,41,87] and somewhat misconstrued. Models of the evolution of female choice have shown that genes with non-additive effects do not contribute to indirect selection on female choice (see Introduction), so any selection on female choice for genetic compatibility benefits must be direct. It is also worth noting that in order for females to gain indirect benefits by choosing compatible mates, females need to assess their own genome, assess the male genome, and predict the genetic compatibility effects on offspring fitness. The latter also requires taking into account recombination, asymmetric inheritance patterns and sex-specific effects [88]. Although this may seem highly unlikely, female choice for compatible males may nevertheless occur for specific loci or more restricted regions of the genome, such as the major histocompatibility complex (MHC) in vertebrates [89].

In general, the presence of genetic variation for fitness has been interpreted as supporting a role for indirect genetic benefits in the evolution of female choice [62]. However, three points call this interpretation into question. First, when indirect selection does occur, the efficacy of indirect selection may nevertheless be low in the face of direct selection [12,14]. Direct selection on female preference is probably generally underestimated in empirical research [90,91]. In our model system, direct selection on female choice is likely to be strong [92,93] and thus may override indirect selection. However, Fricke and Arnqvist (2007) showed that sexual selection accelerated adaptation to a novel environment in *C. maculatus*, suggesting a role for indirect selection under at least some conditions. Second, indirect selection on female choice arises as a result of linkage disequilibrium between female choice/preference genes and genes with additive effects that confer high fitness in offspring [12]. In order for such linkage disequilibrium to build up, even in the presence of additive genetic variation for fitness, females with high level of preference must consistently choose males with a high breeding value for fitness. Very few empirical studies have looked for the consistency of such associations [67]. This problem is aggravated when considering direct selection for indirect benefits in the form of mate compatibility. Here, demonstrations of epistatic fitness variation in conjunction with male × female interactions in female choice are insufficient: females must also be able to consistently bias fertilization success among males towards males that carry genes that are compatible with her own genes. Empirical evidence for such associations are restricted to the study of MHC in vertebrates [89] and some studies of the deleterious effects of inbreeding [94,95].

Third, perhaps the most fundamental problem in assessing the potential for indirect genetic benefits in sexual selection lies in estimating net selection on female choice [90]. In order to measure paternal genetic contribution to fitness it is vital to obtain proper estimates of offspring genetic quality, preferably the total number of grandchildren produced [7,60,96]. Fitness correlates such as fecundity and lifetime offspring production are often reported and assumed to correlate positively with net fitness, but life-history theory and empirical evidence provide little basis for expecting strong positive correlations between individual fitness components and overall genetic quality (i.e., breeding value for fitness) [66-68]. Further, recent empirical research has revealed a negative, or a non-existing, genetic correlation between male and female fitness, suggesting that potential indirect benefits might be nullified through sexually antagonistic genes [60,61,66,97,98]. This points to the necessity of measuring the breeding value for fitness in offspring of both sexes, when assessing the potential role of indirect benefits for the evolution of mating biases. Such investigations are currently in progress in *C. maculatus*.

## Conclusion

In conclusion, we found significant standing genetic variance for fitness related traits in our study population of *C. maculatus*, in sufficient amounts for females to potentially derive indirect genetic benefits of mate choice. The genetic architecture of fitness traits varied dramatically with the life history stage in which fitness traits were measured. Parental effects, in particular paternal effects, were significant in the juvenile stage, while sizeable additive and non-additive effects were seen in the lifetime reproductive success of F_1 _daughters. Our results support the notion that comprehensive fitness measures should include offspring breeding value. Although our results show that there is a potential for females to derive indirect genetic benefits from mate choice, we note that several facts may nevertheless make selection for indirect genetic benefits ineffectual. These include direct selection, lack of accuracy in female choice and sexually antagonistic genetic variation.

## Authors' contributions

TB participated in the design of the study, collected data, performed statistical analyses and wrote the paper. UF and AM participated in the design of the study and collected data. JF participated in the statistical analyses and writing of the paper. GA participated in the design of the study and the writing of the paper. All authors read and approved the final manuscript.
